# Extraction of PE Online Teaching Resources With Positive Psychology Based on Advanced Intelligence Algorithm

**DOI:** 10.3389/fpsyg.2022.948721

**Published:** 2022-07-08

**Authors:** Wei Wang, Juan Hu

**Affiliations:** ^1^Department of Physical Education, Nanjing University of Finance and Economics, Nanjing, China; ^2^Department of Social Sports Guidance and Management, Nanjing Sport Institute, Nanjing, China

**Keywords:** PE online teaching resources, positive psychology, psychology classification, advanced intelligence algorithm, information extraction

## Abstract

Physical education (PE) teaching resources occupy a very important position in the teaching of PE theory. Especially in the context of the Internet era, how to effectively extract PE teaching resources from the Internet is very important for PE teachers. However, the quality of PE teaching resources on the Internet is uneven, if not correctly identified, it will bring harm to students’ values. Therefore, it is very necessary to correctly identify the teaching resources of positive psychology. In the era of artificial intelligence, advanced intelligent algorithms provide a solution for the realization of this purpose. In this study, a text sentiment analysis model multi-layer-attention convolutional neural network (ACNN)-CNN based on hierarchical CNN is proposed, which combines the advantages of convolutional neural networks and the attention mechanism. In multi-layer-ACNN-CNN, position encoding information is added to the embedding layer to improve the accuracy of text sentiment classification. In order to verify the performance of the model, online PE teaching resources are extracted by a crawler system and the proposed model is used to classify the positive psychology of the teaching resources. By comparison, the proposed model obtained a better positive psychology classification effect in the experiment, which verifies that the model can extract text features more accurately, and is more suitable for emotion classification of long texts.

## Introduction

The Ministry of Education of the People’s Republic of China released the “Education Informatization 2.0 Action Plan” on April 13, 2018 (He, 2021; Yan and Yang, 2021). This action plan is an inevitable choice for the development of education in the era of intelligence, and it is a specific plan to promote “Internet + Education.” In addition, in recent years, China has also issued a series of policy documents, which put forward new requirements for the construction of high-level teaching staff in the Internet era. College PE students and PE normal students are the typical teaching execution groups in the field of PE, and they are the reserve talents of teachers in China. However, at present, the single teaching method of PE and the outdated traditional learning forms are still the problems that need to be solved urgently in the training process. In the “Internet +” era, the “Education Informatization 2.0 Action Plan” is an effective way to accelerate the modernization of education. That is to say, using modern Internet information technology, building and upgrading online education platforms, optimizing online PE teaching resources, innovating and improving education and teaching methods to improve students’ learning efficiency and reduce the cost of acquiring knowledge and information, will definitely make a huge contribution to the construction of a high-level PE teaching group. In 2020, COVID-19 broke out around the world, and it still has a huge negative impact on people’s life and learning. It is also a test for teachers (Lu, 2020). Teachers need to better master new media technology and the ability to operate teaching software. Before teaching, teachers should collect a lot of information from the Internet, take the essence and get rid of the dross, and then process it into a lesson plan in the course. PE teachers need to establish fascinating teaching situations and provide students with multi-sensory comprehensive stimulation, which can improve students’ learning enthusiasm and fully arouse students’ interest in learning (Li, 2009). The network environment also provides students with learning resources with a large amount of information, vivid forms, rich contents and interactive functions. Students can obtain learning resources from various channels according to the needs of the discussion topics, which is more in line with the development law of students’ cognitive structure. With its rich resources, the Internet provides a good platform for our education and teaching reform. Therefore, we should make full use of network teaching resources, strengthen the main body of students’ learning, and guide students to conduct independent inquiry learning as the focus of physical education.

Different scholars have different views on the definition of PE teaching resources. Some scholars believe that PE teaching resources refer to all the materials, manpower and information which are useful for PE (Elliot et al., 2013). According to the scope of use, PE teaching resources are divided into six types, namely human resources, sports facilities resources, sports project resources, media resources, extracurricular and extracurricular resources, and natural resources. Environmental resources. In addition, some scholars believe that PE teaching resources are a concept with a wide range of content, which is the sum of all resources that are exposed in the process of PE, including social, school and family resources, as well as the geographical location and environments (Yang, 2021). Therefore, it is a general term for all resources that serve PE. In theory teaching of PE, text resources occupy the dominant position of all resources. The positive emotions contained in text resources of PE have an important influence on the formation of students’ values. Don Hellison, a professor at the University of Illinois, believes that personality training and civic responsibility education are the development goals of PE (Hellison, 2010). Under the background of the increasingly serious personality crisis behavior of students, the moral learning involved in theory courses of PE has also received more and more attention. From current studies, we think that the purpose of PE teaching resources is to provide a kind of information service for teachers and students. This service integrates PE teaching mode, knowledge utilization, teacher-student relationship and so on, and then is embedded in the Internet platform as the carrier of the service.

Therefore, in this study, we take the text resources in PE theory teaching as the research object, and use advanced artificial intelligence methods to analyze the emotion of text resources (Xian, 2010; Yang et al., 2020; Wang, 2021; YanRu, 2021). The quality of PE teaching resources on the Internet is uneven, if not correctly identified, it will bring harm to students’ values. Therefore, it is very necessary to correctly identify the teaching resources of positive emotions. In the era of artificial intelligence, advanced intelligent algorithms provide a solution for the realization of this purpose. Text emotion analysis refers to the use of natural language processing, text analysis and statistical learning to mine and identify the views and opinions contained in raw data (Chaffar and Inkpen, 2011; Ran et al., 2018). Text emotion classification is the core of emotion analysis. In this study, the main contributions can be summarized as: (1) a text emotion analysis model multi-layer-ACNN-CNN based on hierarchical CNN is proposed, which combines the advantages of convolutional neural networks and the attention mechanism. (2) In multi-layer-ACNN-CNN, position encoding information is added to the embedding layer to improve the accuracy of text emotion classification. The following sections are organized as follows. In “Positive Psychology Classification of PE Teaching Resources,” we present our method regarding positive psychology classification of PE teaching resources. In “Experimental Studies on Extraction of PE Online Teaching Resources With Positive Psychology,” we report our experimental results on extraction of PE online teaching resources with positive psychology. In the last section, we conclude the whole study.

## Positive Psychology Classification of PE Teaching Resources

**Figure 1:**
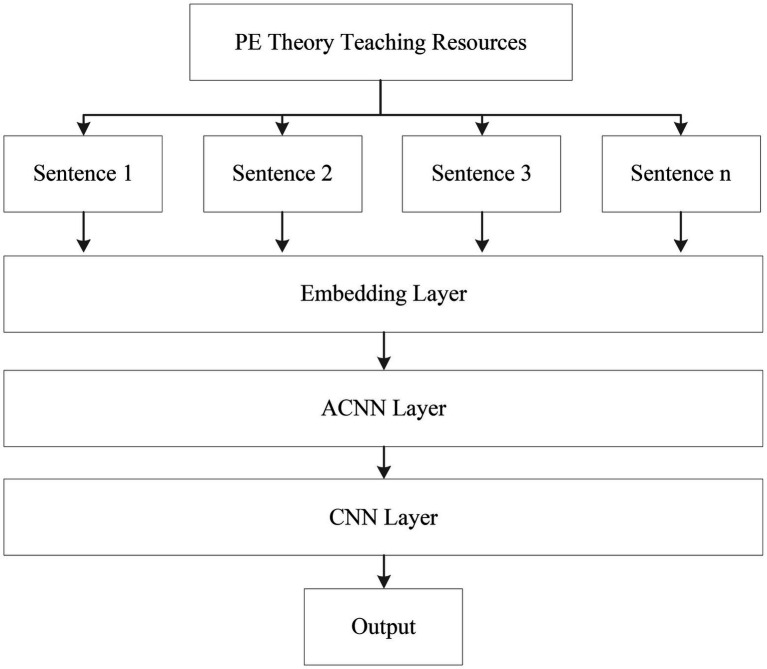
Structure of multi-layer-ACNN-CNN.

To achieve the text emotion classification task of online teaching resources of PE, in this study, based on convolutional neural networks (CNN; Chauhan et al., 2018), a hierarchical long text emotion classification model multi-layer-ACNN-CNN based on the combination of the attention mechanism and CNN is proposed. Multi-layer-ACNN-CNN consists of an embedding layer, an ACNN layer and a CNN layer, where the embedding layer is a word embedding layer with position information, the ACNN layer is composed of CNN based on the attention mechanism, and the last CNN layer is composed of CNN based on the text layer. The structure of multi-layer-ACNN-CNN is shown in Figure 1.

PE online teaching resources usually contain strong emotional tendencies, and there is a causal relationship in expression, so constructing sentence pairs can extract key information. Therefore, in PE teaching resources, the two sentences before and after are formed into a sentence pair. For each sentence, the word vector representation is first obtained through Word2vec (word to vector), and the position encoding information is added according to the word position. ACNN is used to extract the feature information between sentence pairs, and then the feature information of all sentence pairs is input into CNN, and the global features of the entire text are extracted through CNN. Based on the global features, the final emotion classification results can be obtained.

### Word Embedding

There are many ways to represent word vectors, the most commonly used in the past is the bag-of-words model. However, the bag-of-words model does not consider the contextual relationship between words in a sentence, but only considers the weight of the word itself, which is only related to the frequency of the word appearing in the text. On the other hand, the bag-of-words model will have problems such as sparse distribution and high dimension, which is not easy to calculate and requires a lot of computation. In this study, Word2vec is used to obtain the word vector representation (Church, 2017). Word2vec is an open-source tool from Google which combines artificial neural networks and probabilistic models to improve neural language models. Word2vec includes two training models, CBOW and Skip-gram. In this study, we use the CBOW (continuous Bag of Words) model to obtain word vectors as the input of the ACNN layer (Liu, 2020). In this study, we take words as the unit. For a sentence, the sentence vector can be expressed as the result of splicing word vectors.

**Figure 2:**
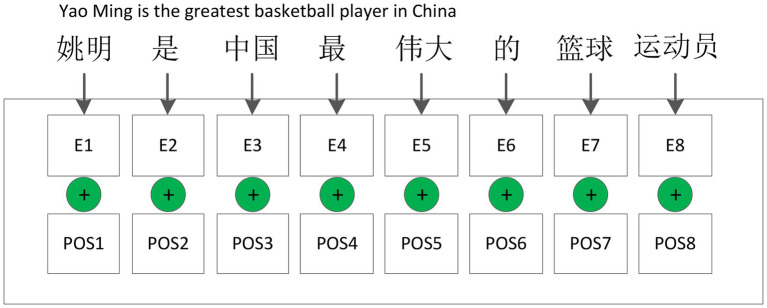
Structure of embedding with position information.

Since the proposed model is based on CNN, the ability to preserve sentence sequence information is not strong (Cheng et al., 2020). The sequence information represents the global structure and is particularly important. Therefore, in order to make full use of the order of the sentence itself, in this study, we added the relative position encoding information of the word in the sentence in each word. There are various ways to construct the position-encoding information function, here we use the sine and cosine functions (Chauhan et al., 2018). This method can be applied to the case where the sentence length in the test set is longer than that in the training set. The structure of the embedding layer with position information is shown in Figure 2. As shown in Figure 2, position information can be computed by the following three equations,


(1) $$PE_{\left(pos,2i\right)}=\sin\left(\frac{pos}{10000^{\frac{2i}{d}}}\right)$$

(2) $$PE_{\left(pos,2i+1\right)}=\cos\left(\frac{pos}{10000^{\frac{2i}{d}}}\right)$$

(3) $$POS=PE_{\left(pos,2i\right)}⊕PE_{\left(pos,2i+1\right)}$$

where *POS* represents the position-encoding information vector of the word, *pos* is the position of the word in the sentence, *d* represents the dimension of the word vector, *i* represents the *i*-th element in the word vector, and ⊕ is the splicing operator. The encoding information has the same dimension as the word embedding vector matrix, which can be directly superimposed and summed for the following computation.

### Attention Convolutional Neural Network

**Figure 3:**
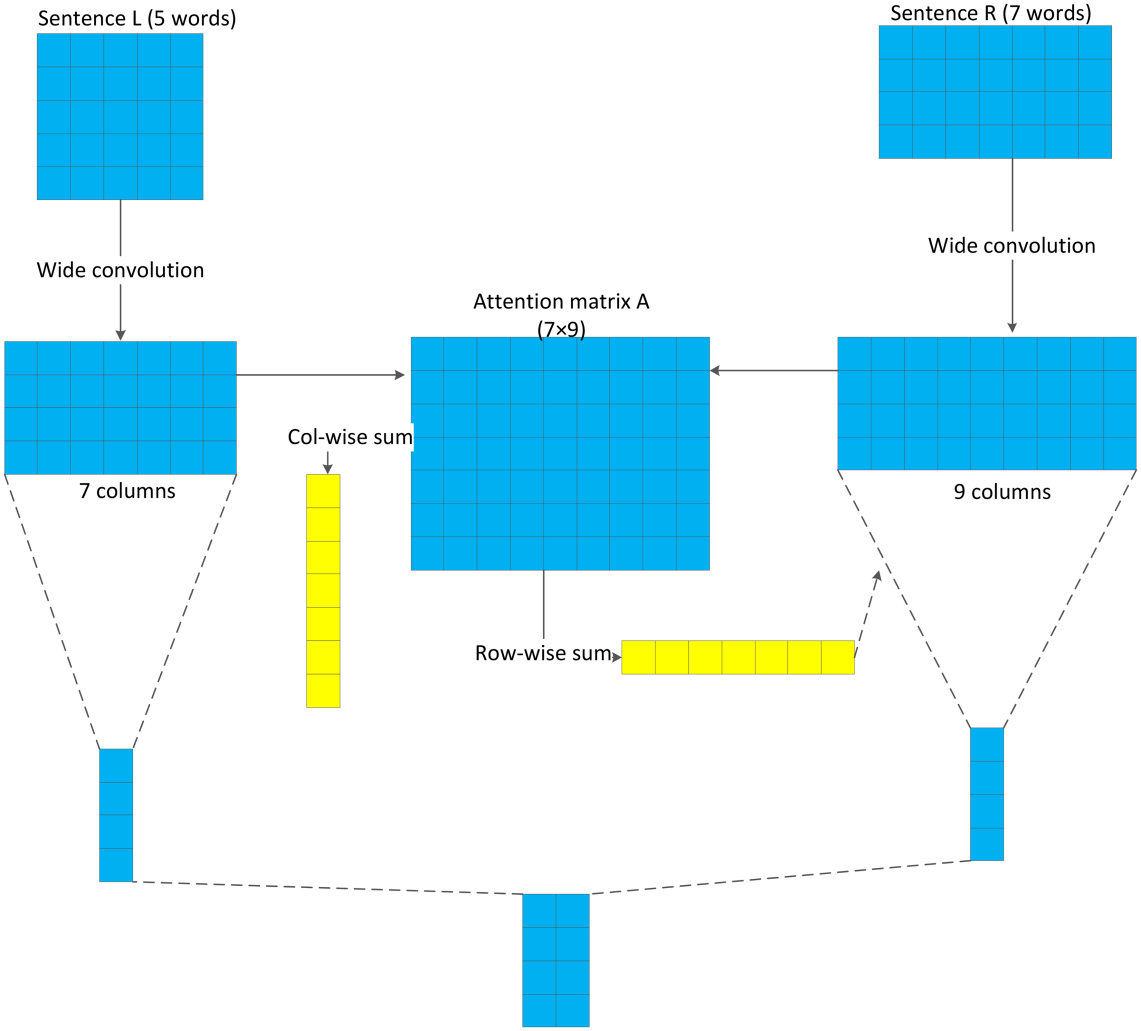
Structure of ACNN.

Below the embedding layer, a convolutional neural network model based on the attention mechanism (Fukui et al., 2019; Yan et al., 2019) termed as Attention Convolutional Neural Network (ACNN) is proposed, as shown in Figure 3. ACNN mainly consists of an input layer, a wide convolutional layer, an attention-based pooling layer, and a pooling layer. The input layer receives the output of the embedding layer, that is, the word vector feature matrix of each word in the sentence pair. The wide convolution uses its own characteristics to perform the convolution operation on the basic unit of the input layer to extract features. The attention mechanism is added to the pooling layer to extract the sentiment polarity between different words. Finally, the feature information of the two sentences is fused through the merge layer to obtain the feature representation vector of the sentence pair.

#### Wide Convolution

Suppose we have a convolution kernel of size *k* and a sentence of length *n*. *L_i_* ∈ *R^d^* is the *d*-dimensional vector representation of the *i*-th word in the sentence, *L*∈*R^n^* × *d* represents the input sentence, and the vector *m*∈ *R^k^* × *d* represents the convolution kernel used in the convolution operation. For the *j*-th position in the sentence, a window vector matrix of the same size can be obtained according to the size of the convolution kernel. It consists of *k* consecutive word vectors that can be computed by


(4) $$w_{j}=\left[L_{j},L_{j+1},\ldots ,L_{j+k-1}\right]$$

The convolution kernel *m* convolves the window vector (k-gram) at each position. The idea behind the one-dimensional convolution is to multiply the elements of the matrix by the convolution kernel *m* and each k-gram in the sentence *L* to obtain the feature map *c_j_* as


(5) $$c_{j}=f\left(m^{T}w_{j}+b\right)$$

where *b* is the bias term, *f(x)* is a nonlinear transformation function, *sigmoid*, *tanh*, *reLU*, etc. In this study, *ReLU* is adopted as the activation function.

When using the traditional convolution kernel to perform convolution operations, it is often impossible to operate on the data at the edge. To solve this problem, in this study, zero-padding is used to construct a wide convolution. Suppose we have *n* input nodes, from *L_1_* to *L_n_*, the size of the convolution kernel *m* is *k*. Through classical convolution operation, the number of input nodes is reduced to *n-k* + 1. Note that There is no corresponding convolution operation for (*k*-1)/2 nodes at the edge, so relevant information about these two nodes will be lost. In this study, we use the zero-padding method, that is first to add (*k*-1)/2 nodes on the edge of the matrix, and then perform the convolution operation to get (*n* + *k*-1) nodes, even larger than the original matrix. We perform a wide convolution operation after the input layer to preserve all the information in the sentence as much as possible to improve the accuracy of the final classification.

#### Pooling Layer Based on Attention Mechanism

In order to make the model distinguish important information during training, in this study, we add an attention mechanism in the pooling layer to make the model pay high attention to this information. In the convolutional layer, we perform a wide convolution operation on two consecutive sentences to obtain two different output vectors, named C_L_ and C_R_. Based on these two vectors, the attention weight vector matrix A can be obtained by


(6) $$A_{i,j}=\mathrm{MatchScore}\left(C_{L}\left[:,i\right],C_{R}\left[:,j\right]\right)$$

where $A_{i,j}$ represents the distance metric between the i-th column vector of C_L_ and the j-th column vector of C_R_. It can be defined as 1/(1 + |C_L_-C_R_|), |C_L_-C_R_| represents the distance measure of the left and right vectors. There are many methods to compute the distance, such as Euclidean distance, cosine similarity, and Manhattan distance. In this study, Euclidean distance is used.

After obtaining the attention weight vector matrix A, we need to calculate the convolution vector weight, and assign a weight to each convolution layer. The attention weight $a_{L,j}$ corresponding to each unit of the left vector is the sum of the column vectors in the weight vector matrix A, which can be computed by


(7) $$a_{L,j}=\sum A\left[j,:\right]$$

Similarity, $a_{R,j}$ can be computed by


(8) $$a_{R,j}=\sum A\left[:,j\right]$$

When pooling, we multiply and sum the output feature matrix after convolution, associating with the weight value of attention, to extract important feature information, and then connect them into a vector to finally obtain the output of the pooling layer.

### CNN Layer

With ACNN, finally, the information features of sentence pairs can be obtained based on attention mechanism convolutional neural network. Suppose we have a certain text with *s* sentences to be classified, the two sentences before and after are organized into a sentence pair, so finally we have *s*-1 sentence pairs. Therefore, through the ACNN layer, we finally obtain s-1 feature vectors. The text vector can be represented as


(9) $$F=P_{1}⊕P_{2}⊕P_{3}\ldots ⊕P_{s-1}$$

The text feature vector *F* is used as the input of the CNN model. The entire CNN model consists of the following 4 parts. The first part is the input layer, which is expressed by *F*. The second layer is convolution layer. In this layer, different convolution kernels are used to extract features from *F*. The third layer is the pooling layer, in which max-pooling is used to get the optimal features. The last layer is the full connection layer which is used to compute the possibility distribution corresponding to the input features. *Softmax* is commonly is used to compute the possibility distribution.

## Experimental Studies on Extraction of PE Online Teaching Resources With Positive Psychology

### PE Online Teaching Resources

We use a crawler system to extract PE online teaching resources from data fountain^1^ and finally we obtain 4,000 text documents. A 20 PE teachers are employed to organized these text documents into 28,400 short texts. In addition, there 20 PE teachers are also invited to manually label 60% of these short texts with positive emotion and negative emotion. Table 1 illustrates a toy example of the labeled short texts, where positive emotion refers the refers to the positive and objective content contained in the corpus. Negative emotion is those in which the corpus contains discouraging or deliberately false information. Among the 60% labeled short texts, 80% of them is used as the training set and 20% of them is used as the validation set.

**Table 1:** Example of PE online teaching resources.

**Text**	**Emotion label**
中国代表团在东京奥运会上获得38枚金牌，32枚银牌，18枚铜牌，4次打破世界记录 (The Chinese delegation won 38 gold medals, 32 silver medals, 18 bronze medals at the Tokyo Olympics, breaking world records 4 times).	Positive
苏炳添在东京奥运会田径男子100米半决赛中跑出9秒83的亚洲最好成绩 (Bingtian Su clocked an Asian best time of 9.83 s in the men’s 100 m semi-finals at the Tokyo Olympics).	Positive
2013年，孙杨在杭州体育馆路口与一辆剬交车发生了刮蹭，虽然是剬交车负全责，但孙杨也被查出了无证驾驶，被国家游泳队处罚 (In 2013, Sun was caught driving without a license and was punished by the national swimming team, although the bus was fully responsible for his collision at the intersection of Hangzhou Stadium).	Negative

### Experimental Settings

To highlight the proposed multi-layer-ACNN-CNN, five external models are introduced for comparison studies, as shown in Table 2.

**Table 2:** Four comparison models.

Models	Descriptions
CNN	Classical convolutional neural network
CNN-CNN	Two-layer classical convolutional neural network
ACNN	Convolutional neural network with attention mechanism
Position-ACNN	Convolutional neural network with attention mechanism and position information

During the training process, Adam is used to optimize the model, and the activation function of the convolutional layer is ReLU. Other settings of specific parameters are shown in Table 3.

**Table 3:** Model parameter settings.

Parameters	Descriptions	Values
m	Kernel size	3, 4, 5
n	Number of kernels	128
p	Dropout rate	0.5
b	Batch size	64

We also introduce Precision (P), Recall (R) and Accuracy as evaluation criteria to evaluate the performance of all models. P, R and Accuracy are defined as follows,


(10) $$P=\frac{TP}{TP+FP}$$

(11) $$R=\frac{TP}{TP+FN}$$

(12) $$\mathrm{Accuracy}=\frac{2\times P\times R}{P+R}$$

where *TP* is the number of samples whose real category is positive and predicted to be positive; *FN* is the number of samples with positive real category and negative predicted category; *FP* refers to that the number of samples with negative real category and positive predicted category; *TN* is the number of samples with negative real category and negative predicted category.

All experiments are conducted on a PC with i7-11800H CPU, GTX-2080 Ti 12G GPU, and 64G memory.

### Experimental Result Analysis

**Figure 4:**
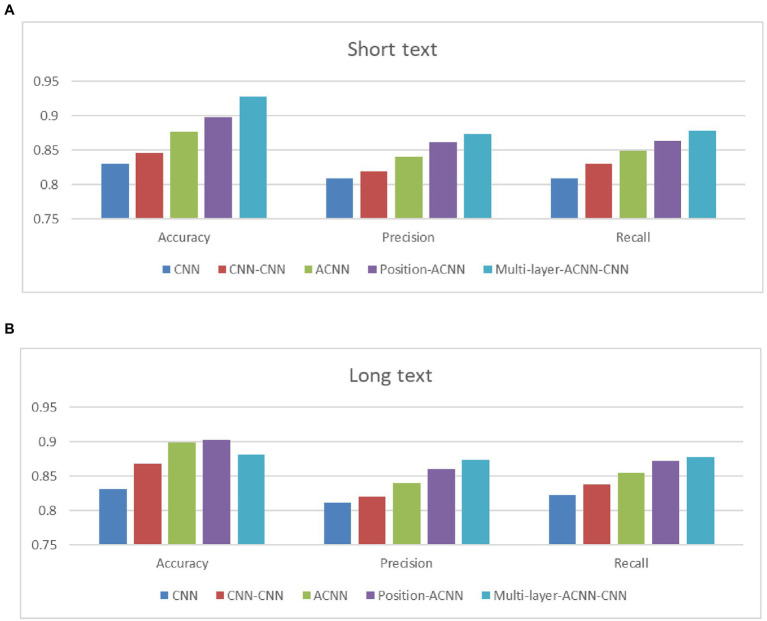
Emotion classification comparison in term of Accuracy, P and R. **(A)** Short text and **(B)** long text.

As can be seen from Table 4 that the overall deep learning-based model has achieved good classification results on PE teaching resources, and the Multi-layer-ACNN-CN model proposed in this study has the best performance in terms of Accuracy, P and R (Figure 4).

**Table 4:** Emotion classification results in term of Accuracy, P and R.

Models	Accuracy		Precision		Recall	
	Short text	Long text	Short text	Long text	Short text	Long text
CNN	0.8298	0.8312	0.8096	0.8111	0.8098	0.8231
CNN-CNN	0.8456	0.8678	0.8197	0.8200	0.8301	0.8378
ACNN	0.8765	0.8987	0.8398	0.8403	0.8487	0.8544
Position-ACNN	0.8982	**0.9021**	0.8611	0.8599	0.8642	0.8721
Multi-layer-ACNN-CNN	**0.9283**	**0.8899**	**0.8732**	**0.8811**	**0.8785**	**0.8890**

*Bold values mean the best performance*.

Comparing with the CNN and ACNN models, it can be seen that the performance of ACNN is far better than that of CNN. This is because the attention mechanism compares and analyzes the importance of each word in the text, so it can better grasp the impact of key words in the text on the text’s emotional tendency, thereby improving the accuracy of sentiment classification. Comparing with the CNN and CNN-CNN models, it can be seen that the performance of the multi-layer model is better, indicating that the complex model has more advantages in the face of a large amount of data. Multi-layer models can better extract feature information from long texts. Comparing the three models of ACNN, position-ACNN and Multi-layer-ACNN-CNN, it can be seen that position-ACNN has slightly improved performance compared with ACNN, because Multi-layer-ACNN-CNN adds position coding information and retains words’ location information, so its accuracy is higher. What is more, Multi-layer-ACNN-CNN adds a layer of CNN on the basis of pos-ACNN, which can better apply to complex data sets.

On the other hand, comparing the performance of all models on short and long texts, it is clear that the single-layer model performs better on short texts, but not on long texts. The performance of Multi-layer-ACNN-CNN proposed in this study on long texts is basically the same as that on short texts in terms of precision and accuracy, while the recall rate is 0.0093 higher, which verifies that position-applicability of the ACNN-CNN model on long texts. On the one hand, the complex hierarchical model is more suitable for feature extraction of long texts, and on the other hand, the application of attention mechanism and the combination of location information make the extracted features more accurate and more able to reflect the emotional tendencies of the text.

## Conclusion

In this study, a text emotion classification model based on hierarchical CNN is proposed, which combines the model advantages of convolutional neural networks, and captures the important features of text through the attention mechanism, and at the same time adds position encoding information to the embedding layer, thereby improving the accuracy of text classification. In order to verify the performance of the model, it is tested on PE teaching resources, and multiple sets of comparative experiments are designed. The model obtained a better classification effect in the experiment, which verified that the model can extract text features more accurately, and is more suitable for emotion classification of long texts.

In the future work, the hierarchical attention mechanism will be considered, and the ordinary text emotion classification will be split into the classification from the sentence layer and the classification from the text layer, and combined with sequence information models such as LSTM to explore various combinations.

## Data Availability Statement

Publicly available datasets were analyzed in this study. This data can be found at: https://www.datafountain.cn/datasets.

## Ethics Statement

The studies were reviewed and approved by the Ethics Committee of Nanjing University of Finance and Economics and Nanjing Sport Institute. The participants provided their written informed consent to participate in this study. Written informed consent was not required for this study in accordance with the national legislation and the institutional.

## Author Contributions

WW contributed to writing and data collection. JH contributed to data preprocessing. All authors contributed to the article and approved the submitted version.

## Conflict of Interest

The authors declare that the research was conducted in the absence of any commercial or financial relationships that could be construed as a potential conflict of interest.

## Publisher’s Note

All claims expressed in this article are solely those of the authors and do not necessarily represent those of their affiliated organizations, or those of the publisher, the editors and the reviewers. Any product that may be evaluated in this article, or claim that may be made by its manufacturer, is not guaranteed or endorsed by the publisher.

## Footnotes

^1^ https://www.datafountain.cn/datasets
